# Detection of clinically relevant copy number alterations in oral cancer progression using multiplexed droplet digital PCR

**DOI:** 10.1038/s41598-017-11201-4

**Published:** 2017-09-19

**Authors:** Curtis B. Hughesman, X. J. David Lu, Kelly Y. P. Liu, Yuqi Zhu, Rebecca M. Towle, Charles Haynes, Catherine F. Poh

**Affiliations:** 1 Department of Oral Medical and Biological Sciences, Faculty of Dentistry, University of British Columbia, Vancouver, British Columbia V6T 1Z3 Canada; 2 Michael Smith Laboratories, University of British Columbia, Vancouver, British Columbia V6T 1Z4 Canada; 3 Department of Integrative Oncology, British Columbia Cancer Research Centre, Vancouver, British Columbia V5Z 1L3 Canada; 4 Department of Pathology and Laboratory Medicine, University of British Columbia, Vancouver, British Columbia V6T 2B5 Canada

## Abstract

Copy number alterations (CNAs), a common genomic event during carcinogenesis, are known to affect a large fraction of the genome. Common recurrent gains or losses of specific chromosomal regions occur at frequencies that they may be considered distinctive features of tumoral cells. Here we introduce a novel multiplexed droplet digital PCR (ddPCR) assay capable of detecting recurrent CNAs that drive tumorigenesis of oral squamous cell carcinoma. Applied to DNA extracted from oral cell lines and clinical samples of various disease stages, we found good agreement between CNAs detected by our ddPCR assay with those previously reported using comparative genomic hybridization or single nucleotide polymorphism arrays. Furthermore, we demonstrate that the ability to target specific locations of the genome permits detection of clinically relevant oncogenic events such as small, submicroscopic homozygous deletions. Additional capabilities of the multiplexed ddPCR assay include the ability to infer ploidy level, quantify the change in copy number of target loci with high-level gains, and simultaneously assess the status and viral load for high-risk human papillomavirus types 16 and 18. This novel multiplexed ddPCR assay therefore may have clinical value in differentiating between benign oral lesions from those that are at risk of progressing to oral cancer.

## Introduction

Copy number alterations (CNAs), somatic changes involving the gain or loss of genomic material, are common drivers for tumorigenesis^1,2^. CNAs can range in size from microscopic alterations, including deletion of chromosomal segments or entire chromosomes, to submicroscopic alterations where up to a 500 kbp segment of genomic DNA is gained or lost^3^. The importance of CNAs in tumorigenesis has driven extensive studies, including those comprising The Cancer Genome Atlas (TCGA), a genomic repository that includes over 10,000 tumors for which CNAs and other oncogenetic data have been collected (http://www.broadinstitute.org/tcga/home). Sorted by cancer type, TCGA data reveal patterns of common recurrent CNAs that can be used to identify specific loci and larger chromosomal regions more susceptible to alteration in a given cancer^4,5^. Reasons for higher alteration rates in specific genomic regions include the presence of fragile sites^6^ and/or selective pressures promoting oncogene activation or tumor suppressor gene (TSG) inactivation via copy number gains or losses, respectively^4^. For example, focal CNAs involving gain of *CCND1* at 11q13.3 or loss of *CDKN2A* at 9p21.3 are observed in a number of cancers, including oral squamous cell carcinoma (OSCC)^7–9^. But as noted above, specific cancers and their progression are also associated with broader gains or losses of genomic material. One such example in OSCC is a broad gain at the subtelomeric region of 3q that contains many putative oncogenes^8,9^.

The detection of one or more common recurrent CNAs has therefore been considered as a means to assess predisposition toward tumorigenesis^10,11^. Clinically, this concept could be particularly useful in characterizing tissue where histo-pathological assessment alone is not sufficient to reliably predict malignant potential^12^. For example, detection of gains in one or more oncogenes in histologically normal tissue taken from an uninvolved margin of breast cancer has shown promise in defining risk of sporadic breast cancer^11^, while loss of one or more TSGs correlates with risk of oral lesions progressing to OSCC^10^.

Currently, comparative genomic hybridization (CGH) arrays^13,14^, single nucleotide polymorphism (SNP) arrays^15,16^ and whole-genome next generation sequencing (WG-NGS)^17^ are most widely used for genome-wide detection of CNAs. Alternatively, established methods for detecting CNAs at specific targeted genomic loci include fluorescence *in-situ* hybridization (FISH)^18^, multiplex ligation-dependent probe amplification (MLPA)^19^, targeted NGS^20–22^, and quantitative PCR (qPCR)^23^. An emerging technique capable of quantitative multiplex detection of CNAs is digital PCR^21–24^. Digital PCR has a number of specific attributes, including low cost and high sensitivity, that make it amenable and particularly attractive for routine clinical use^25^.

We have previously described a general strategy for designing and analyzing multiplexed droplet digital PCR (ddPCR) experiments to accurately quantify CNAs in genomic DNA recovered from tissue samples^26^. In this study, we expand on that work to develop a novel multiplexed ddPCR assay designed to detect CNAs and larger chromosomal alterations that have been frequently observed to occur and/or to drive progression from oral precancerous lesions to OSCC. The method targets loci associated with common recurrent CNAs in OSCC progression found in the chromosomal regions 3p, 3q, 4q, 5p, 7p, 8p, 8q, 9p, 11q, 18q, 20p and 20q. Primers targeting the E6 regions of human papillomavirus (HPV) types 16 and 18 are also included^27^, allowing us to concurrently assess for status and viral load of high-risk HPV, an emerging risk factor associated with head and neck squamous cell carcinoma (HNSCC), especially at the oropharyngeal site^28–30^. We demonstrate that this ddPCR-based assay detects CNAs at the targeted loci in a manner that is consistent with data from benchmark methods (CGH arrays^31–34^, SNP arrays^35^ and WG-NGS^36^) for both oral cell lines and clinical samples of different histopathological stages. We demonstrate advantages of the multiplexed ddPCR assay relative to these benchmark methodologies used to detect CNAs genome-wide, including the ability to detect and quantify submicroscopic homozygous deletions (HDs). Further, we show that the new method can infer the average ploidy level, from which viral loads and high-level copy number gains can be determined. Finally, the ability of the method to quantify different patterns of CNAs in various histopathological stages is demonstrated through application to cell lines having normal, dysplasia and OSCC phenotypes, and to clinical samples classified as non-progressing and progressing low-grade dysplasia (LGD), high-grade dysplasia (HGD), and OSCC.

## Results

### CNAs detected by our ddPCR Assay in normal, dysplastic and OSCC cell lines

To evaluate the performance of our multiplexed ddPCR assay to detect CNAs, we first analyzed a panel of genomic DNA samples taken from immortalized cell lines representing normal, dysplasia and OSCC phenotypes. Using our previously described sample-specific clustering method^26^, for each cell line tested, a significant number of 13 reference loci were found to be stable, ranging from all 13 loci for the normal and dysplasia cell lines, as well as for one of the OSCC (CAL 27) cell lines, to 7 loci for the SCC-25 OSCC cell line. For each sample, the subset of stable reference loci are used to define a CNA-neutral benchmark as described previously by our group^26^. That in turn allows CNAs, expressed as ${R}_{i/b}^{Norm}$ values where *R* is the normalized ratio of copies of target loci *i* relative to the average copy number of the benchmark, to be calculated, in this case for each of the 24 target loci.

For the OKF4 E6E7 (normal phenotype) cell line, no significant CNAs were detected in the set of target loci, consistent with orthogonal CGH array data (Supplementary Figure S2A). CNAs identified in the dysplastic cell line POE9n tert included an HD at *CDKN2A* and a low-level gain at 5p (*TERT*) (Supplementary Figure S2B). Deletions at *CDKN2A* in POE9n tert have been reported^37^, while gains at 5p are consistent with the previously reported increase in *TERT* expression for this cell line^34,37^.

**Figure 1:**
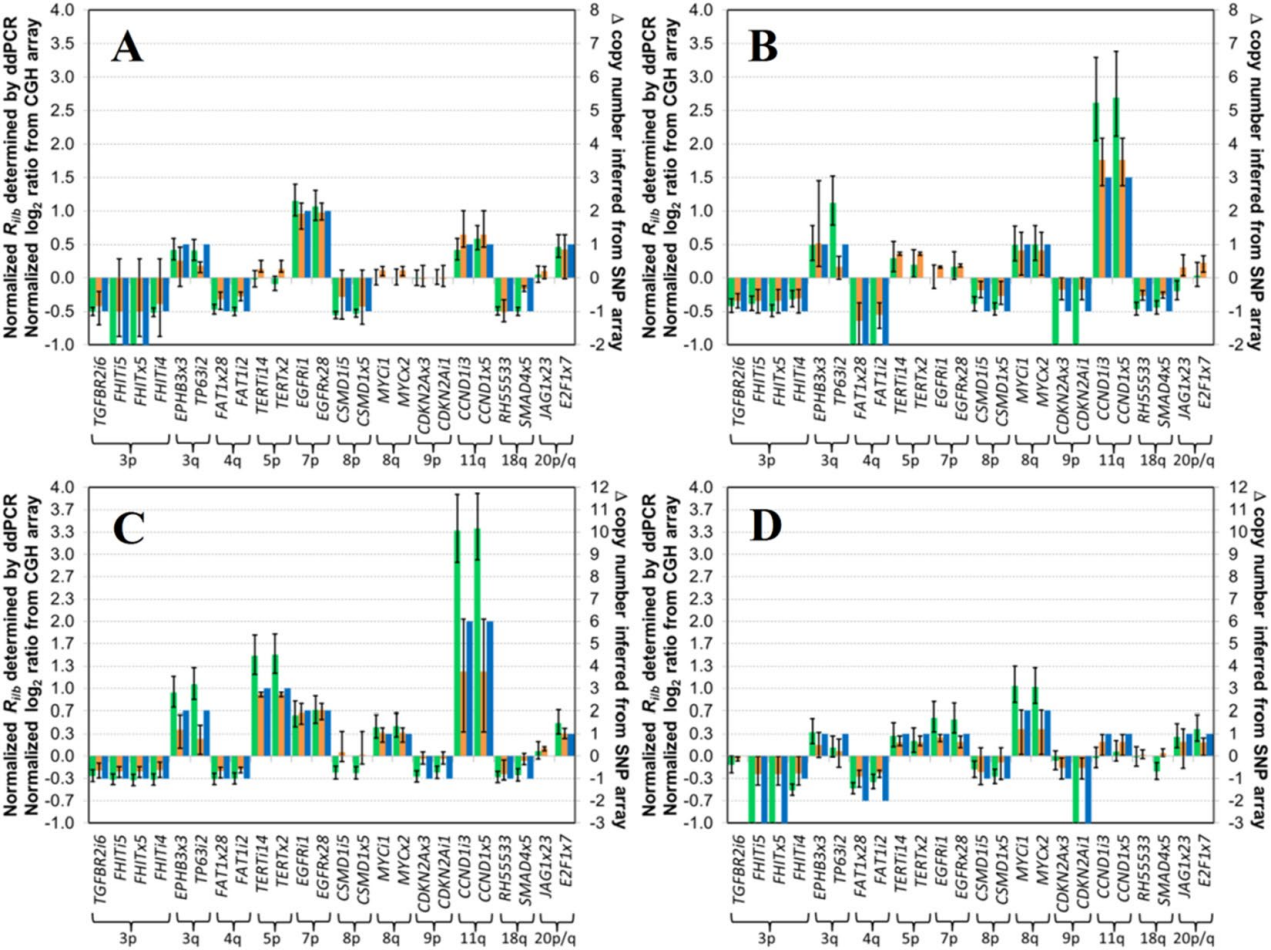
Comparison of CNAs for 24 target loci in cell lines representing different stages of OSCC determined by ddPCR, CGH arrays and SNP arrays. CNAs determined by ddPCR (green bars), array CGH (orange bars) or SNP array (blue bars) for (**A**) CAL27 (inferred to be diploid), (**B**) SCC-25 (diploid), (**C**) SCC-4 (triploid) and (**D**) SCC-9 (triploid). Error bars for ddPCR data represent a 99% confidence interval, while error bars for CGH show the respective high and low normalized log_2_ ratio for the probes that map closest to that target locus. The scale for Δ copy number inferred from SNP array has been adjusted to reflect the ploidy level of the cell lines.

**Figure 2:**
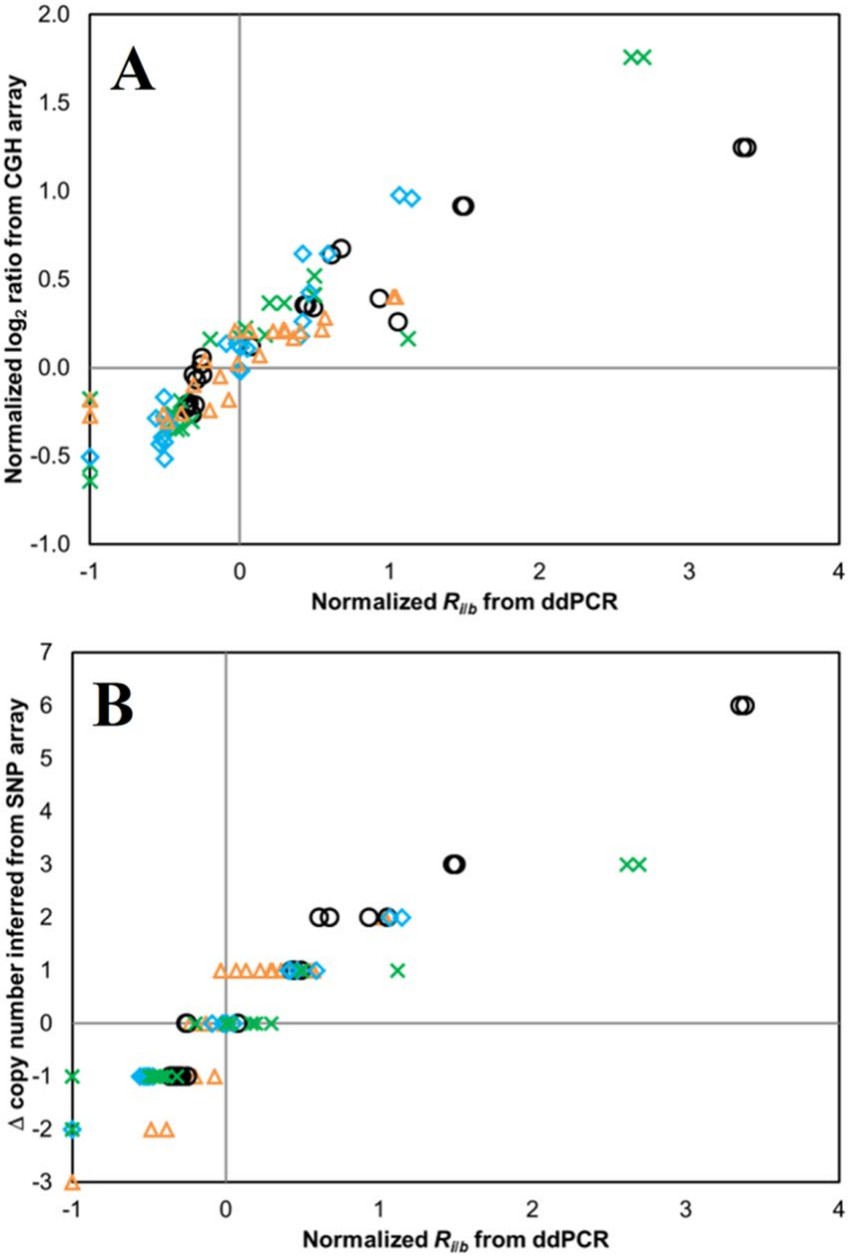
Comparison of CNAs for 24 target loci determined by (**A)** ddPCR *versus* CGH arrays, and (**B)** ddPCR *versus* SNP arrays for the 4 OSCC cell lines studied. CAL27, blue diamond; SCC-25, green x; SCC-4, black circle; SCC-9, orange triangle.

Most of the 24 target loci had statistically significant CNAs in the OSCC cell lines, ranging from 17 CNAs for CAL27 to 23 CNAs for SCC-4 (Fig. 1). Each of these OSCC cell lines has been previously analyzed using CGH or SNP arrays, providing benchmarks for evaluating the performance of our ddPCR assay. For each cell line, the correlation coefficient (*R*) between each pair of analysis methods was determined (Table 1), with combined *R* values for the 4 OSCC cell lines determined to be 0.92 (ddPCR *versus* CGH array), 0.95 (ddPCR *versus* SNP array), and 0.88 (CGH array *versus* SNP array). Importantly, good agreement in both gains and losses at the different target loci was observed between the ddPCR assay and either CGH array (Fig. 2A) or SNP array data sets (Fig. 2B). We have also observed our assay can detect and quantify key genetic events, including HDs, changes in ploidy level, and high-level gains, as well as HPV16 and 18.

**Table 1:** Comparison of oncogenic events and CNAs detected by ddPCR, CGH array or SNP array.

Sample	Ploidy^*1*^		HD^*2*^		High-level Gains^*3*^			Correlation Coefficient (*R*)		
	ddPCR	SNP	ddPCR	SNP	ddPCR	CGH	SNP	ddPCR *versus* CGH	ddPCR *versus* SNP	CGH *versus* SNP
OKF4 E6E7	—	—						*	N/A	N/A
POE9n tert	—	—	9p	N/A				*	N/A	N/A
CAL27	2	1.94	3p	3p	7p	7p	7p	0.96	1.00	1.00
SCC-25	2	1.86	4q/9p	4q	11q	11q	11q	0.95	0.96	0.96
SCC-4	3	2.95			5p/11q	5p/11q	5p/11q	0.93	0.98	0.98
SCC-9	3	3.14	3p/9p	3p/9p	8q		8q	0.89	0.93	0.93
SiHa	3	2.95	3p	3p	5p/20q		5p/20q	0.92	0.94	0.94
HeLa	3	3.46			5p	5p	5p/9p	0.97	0.86	0.86
Oral 67								*		
Oral 48					7p	7p	N/A	0.97		
Oral 44					7p	7p	N/A	0.88		
Oral 75					7p	7p	N/A	0.90		
Oral 91								0.78		

^1^Ploidy level inferred by ddPCR determined using algorithm described in Material and Methods, while inferred ploidy by SNP array is the genome average copy number reported by canSAR.

^2^HD detected by ddPCR or SNP array, no HD was detected by CGH array.

^3^High-level gains were identified as ${R}_{i/b}^{{Norm}}$ or log_2_ value ≥1.0 for ddPCR or CGH array respectively, and were identified as Δ copy number ≥2 for diploid cell lines or ≥3 for triploid cell lines in data from SNP arrays.

^*^ *R* values were not determined for cell lines or clinical samples with few or no significant CNAs.

N/A SNP data not available.

### Detection of HDs

HDs within fragile sites (e.g. *FRA3B*, *FRA16D*) and tumor suppressor genes (e.g., *FAT1*, *CDKN2A*) are known to occur in HNSCCs, including OSCC. Accordingly, three regions of HD were identified by our ddPCR assay in the OSCC cell lines, including deletion of exon 5 of *FHIT* within the *FRA3B* region (3p14.2), deletion of *FAT1* located near the telomeric end of 4q, and deletion of all or part of *CDKN2A* at 9p21.3. Each of these HDs was orthogonally confirmed by SNP array data (Table 1) except for *CDKN2A* observed in the SCC-25 cell line, which had been previously confirmed by homozygous deletion scanning^38^. None of these HDs were detected in the CGH arrays (Table 1).

With additional target loci used at either 3p14.2 or 9p21.1, our assay was able to determine the size of the HD regions (Table 2). HD at *FRA3B* was observed in three target loci in both the CAL27 and SCC-9 cell lines, with the size of the HD thereby determined to be between 12.8 and 551.1 kbp (Table 2). In the three cell lines (POE9n tert, SCC-25 and SCC-9) with known HDs at 9p21.3, we were able to quantify the size of HDs to be between 6.9 kbp and 285.3 kbp (Table 2), encompassing the target loci situated in exon and intron 1 of *CDKN2A*. The size of the HDs at 3p14.2 and 9p21.3 would classify them as being submicroscopic and as noted here were not detected by CGH arrays. Although submicroscopic HDs are generally observed by SNP arrays, very small HDs, such as the one at *CDKN2A* in the SCC-25 cell line, can go undetected.

**Table 2:** Mapped regions of HDs at *FRA3B* 3p14.2 and/or *CDKN2A* 9p21.1 in oral dysplasia and SCC cell lines.

	Target	Start (bp)*	End (bp)*	POE9n tert	SCC9	SCC25	CAL27
*FRA3B (3p14.2)*	*FHITx6*	60,014,021	60,014,113	**×**	**×**	**×**	**×**
	*D3S1234*	60,121,656	60,121,766	**×**	**×**	**×**	**×**
	*D3S1300*	60,524,174	60,524,420	**×**	○	**×**	○
	*FHITi5*	60,524,283	60,524,437	**×**	○	**×**	○
	*FHITx5*	60,536,863	60,536,970	**×**	○	**×**	○
	*FHITi4*	60,672,848	60,672,946	**×**	**×**	**×**	**×**
*CDKN2A (9p21.3)*	*D9S1749*	21,802,946	21,803,065	**×**	**×**	**×**	**×**
	*CDKN2Ax3*	21,967,820	21,967,923	○	**×**	○	**×**
	*CDKN2Ai1*	21,974,618	21,974,715	○	○	○	**×**
	*CDKN2Ax1*	21,974,682	21,974,801	○	○	○	**×**
	*D9S1748*	21,993,776	21,993,895	○	○	**×**	**×**
	*D9S1814M*	22,088,385	22,088,487	**×**	**×**	**×**	**×**

^○^HD (No ddPCR signal detected for target locus).

^**×**^Significant ddPCR signal detected for target locus (and hence absence of a HD).

*Start and end of target amplicon based on GRCh38/hg38.

### Inferring ploidy level

**Figure 3:**
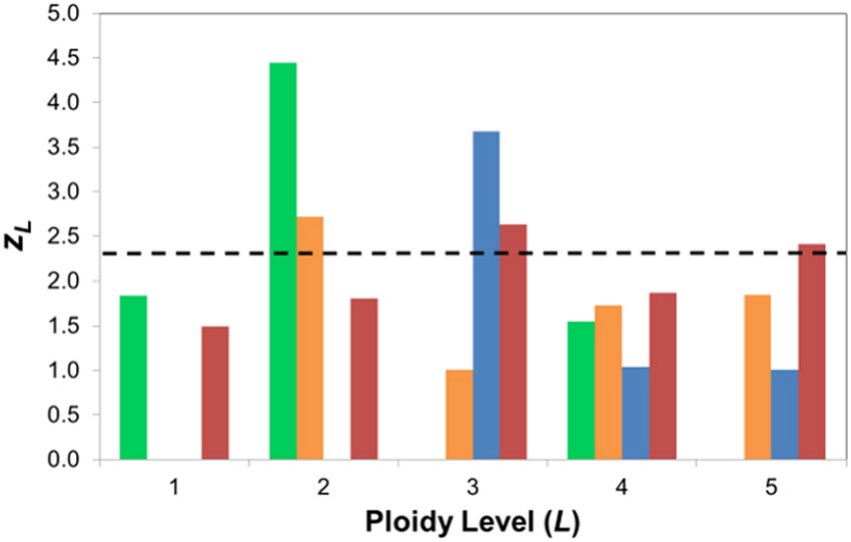
Results of algorithm used to infer ploidy level in the 4 OSCC cell lines. The true ploidy level was taken as that displaying a maximum *z*_*L*_ value. The cell lines CAL27 (green) and SCC25 (orange) are thereby inferred to be diploid, while SCC4 (blue) and SCC9 (red) are inferred triploid with *z*_*L*_ values > 99% significance threshold (dashed line).

Our multiplexed ddPCR assay can measure absolute copy number ratios (*R*_*i/b*_) to infer ploidy level. When applied the data analysis algorithm described in the Methods section for the 4 OSCC cell lines, a diploid status is detected at a 99% significance threshold for CAL27 and SCC-25, and a triploid status is detected for SCC-4 and SCC-9 (Fig. 3); these ploidy levels agree with the average genomic copy number determined by SNP array (Table 1).

### Quantification of high-level copy number gains

Current array CGH and SNP array platforms can be used to detect high-level gains. With SNP arrays, however, accurate estimation of the copy number in regions with high-level gains is challenged by signal saturation effects created when PCR amplification of the sample is conducted. Some algorithms used to interpret raw SNP array data^39^ therefore apply a predefined maximum copy number call, while large copy number call made by alternative algorithms that employ no predefined maximum carry relatively large uncertainties due to the saturation effects observed during DNA hybridization and scanning of array signals^40^. Likewise, uncertainties in array-CGH derived log_2_ values increase, especially for high-level gains.

Our ddPCR-based assay can determine the absolute concentration of amplifiable targets, even when present at relatively high concentrations^41^, to enable accurate quantification of high-level gains. In the SCC-25 cell line, target loci at 11q13.3 (*CCND1i3* and *CCND1x5*) showed CNAs of *R*_*i/b*_ = 3.6 ± 0.6 and 3.7 ± 0.6 respectively, indicating a ~4 fold increase in genomic copies of *CCND1*, which is consistent with previous independent and orthogonal studies reporting elevated *CCND1* copy number in this cell line^42,43^.

### Quantification of HPV 16/18 viral load and detection of CNAs in high-risk HPV-positive cell lines

High-risk strains 16 and 18 of human papillomavirus (HPV) are established prognostic biomarkers for OSCC, with HPV 16/18 infections of the oropharynx accounting for ~20% of all HNSCCs^29^. Our multiplexed ddPCR assay therefore amplifies serotype-specific GP5+/GP6+ targets (~150 bp) within the E6 gene encoded on the long control region of high-risk HPV strains 16 and 18^27^ in a manner that allows one to detect those virulent strains and estimate viral loads in transfected cells or tissue samples. For example, OKF4 E6E7, an immortalized cell line transfected with HPV16 E6E7, tests positive for HPV16, with a *R*_*i/b*_ = 0.6 (±0.1) (Figure S3). Likewise, two additional HPV positive cell lines, SiHa and HeLa, tested positive for HPV-16 and HPV-18, respectively. Using the algorithm described in the Methods section, the inferred ploidy level for both of these cell lines was triploid, consistent with both SNP array data (Table 1) and other studies^36,44^. Multiplying the ploidy level by the copy number ratio *R*_*i/b*_ (where *i* = the HPV-16 or HPV-18 biomarker) allows for estimates of viral load, which for SiHa and HeLa were thereby determined to be 5 (±1) and 13 (±1) copies/cell, respectively (Figure S3), consistent with previously reported viral loads for these non-OSCC cancer cell lines^44,45^. CNAs for the remaining target loci within the assay were also measured. For both SiHa (Figure S4) and HeLa (Figure S5), good agreement was also observed between the ddPCR assay results and corresponding CGH and SNP array data sets (Table 1). Moreover, CNAs within the HeLa cells have also been measured by WG-NGS^36^ (Figure S5), and our ddPCR data correlate well (*R* = 0.98 (ddPCR *versus* WG-NGS)) with that benchmark method, confirming the ability of the multiplexed ddPCR method to quantify CNAs in accordance with the three primary genome-wide CNA-detection methods currently employed in research laboratories – CGH arrays, SNP arrays, and WG-NGS – while offering at low cost a higher throughput than can be realized with any of those benchmarks.

### CNAs detected by our ddPCR assay in clinical tissues representing different histopathological stages

Accurate detection of CNAs in clinical samples is intrinsically challenged by tumor heterogeneity, potential degradation of genomic DNA, and other artifacts, especially within FFPE tissue blocks. Any added uncertainty in assay performance when applied to clinical samples was therefore evaluated by comparing assay results for normal tissue collected from non-diseased patients in the form of fresh blood, frozen tissue block or formalin-fixed paraffin embedded (FFPE) tissue block samples. CNAs were measured for each sample and compared both within and across sample types. DNA extracted from the blood and frozen tissue samples was found to be of high quality with minimal fragmentation. As expected for these normal tissue samples, no statistically significant CNAs were detected at any of the target loci. DNA extracted from FFPE tissue was significantly fragmented; our previously described algorithm^26^ to correct for amplicon-length mediated bias in CNAs was therefore applied. No statistically significant CNAs were then detected for any of the target loci (Figure S6).

**Figure 4:**
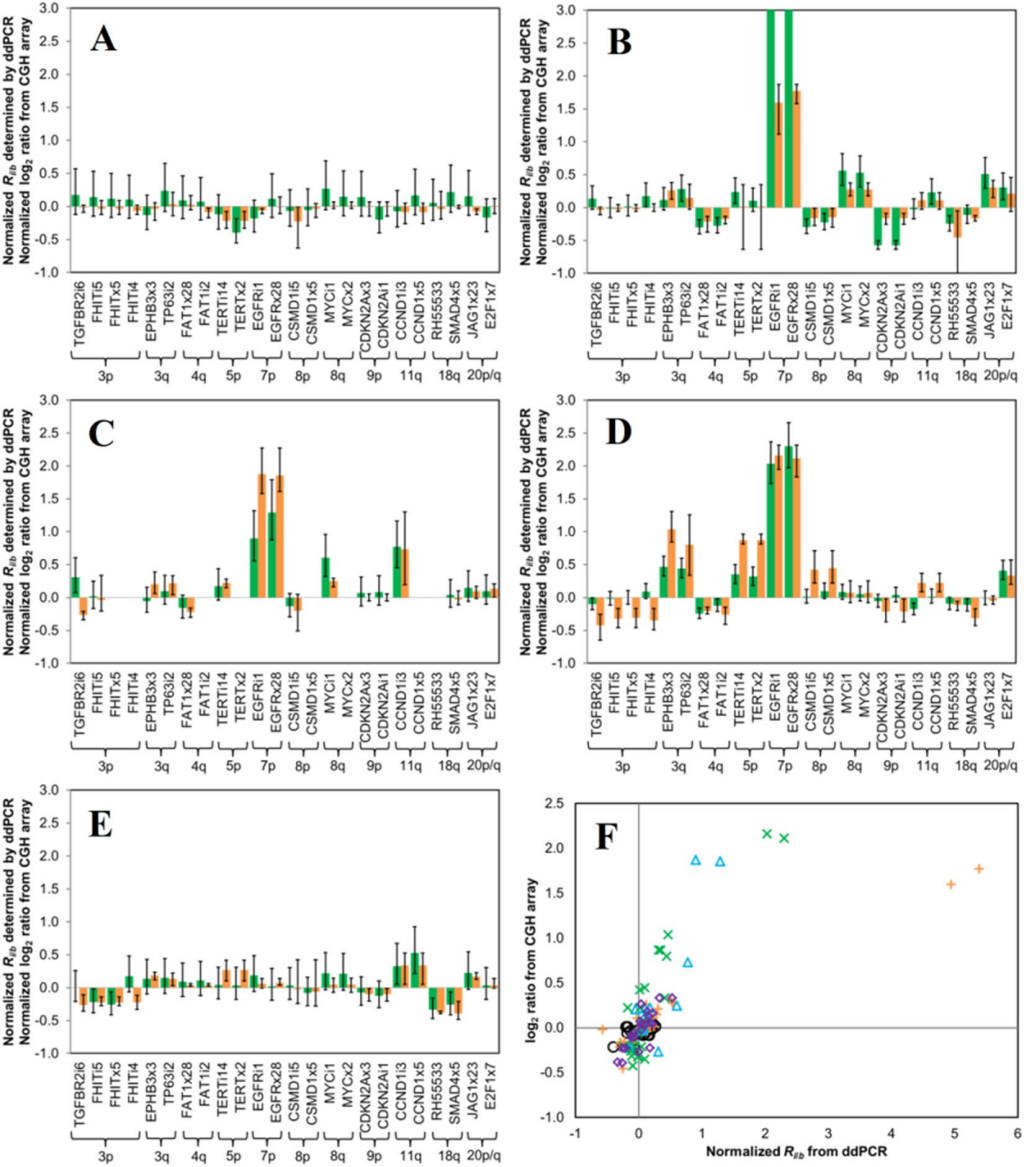
Comparison of CNAs for 24 target loci determined by ddPCR and array CGH for DNA from clinical samples. CNAs determined by ddPCR (green bars) or CGH array (orange bars) for (**A**) non-progressing LGD Oral 67, (**B**) progressing LGD Oral 48, (**C**) HGD Oral 44, and the OSCC samples (**D**) Oral 75 and (**E**) Oral 91. (**F**) Overall correlation of CNAs determined by ddPCR *versus* array CGH for clinical samples Oral 67 (○), Oral 48 (+), Oral 44 (Δ), Oral 75 (x) and Oral 91 (◊). For Oral 44, only 12 of the 16 unique ddPCR reactions were conducted, as this sample had limited DNA available for testing (~200 ng). Oral 48, the *R*_*i/b*_ values for the high-level gains at the target loci *EGFRi1* and *EGFRx28* were 4.9 ± 0.9 and 5.4 ± 1.0 respectively. Error bars for ddPCR data represent a 99% confidence interval, while error bars for CGH show the respective high and low normalized log_2_ ratio for the probes that map closest to that target locus.

**Figure 5:**
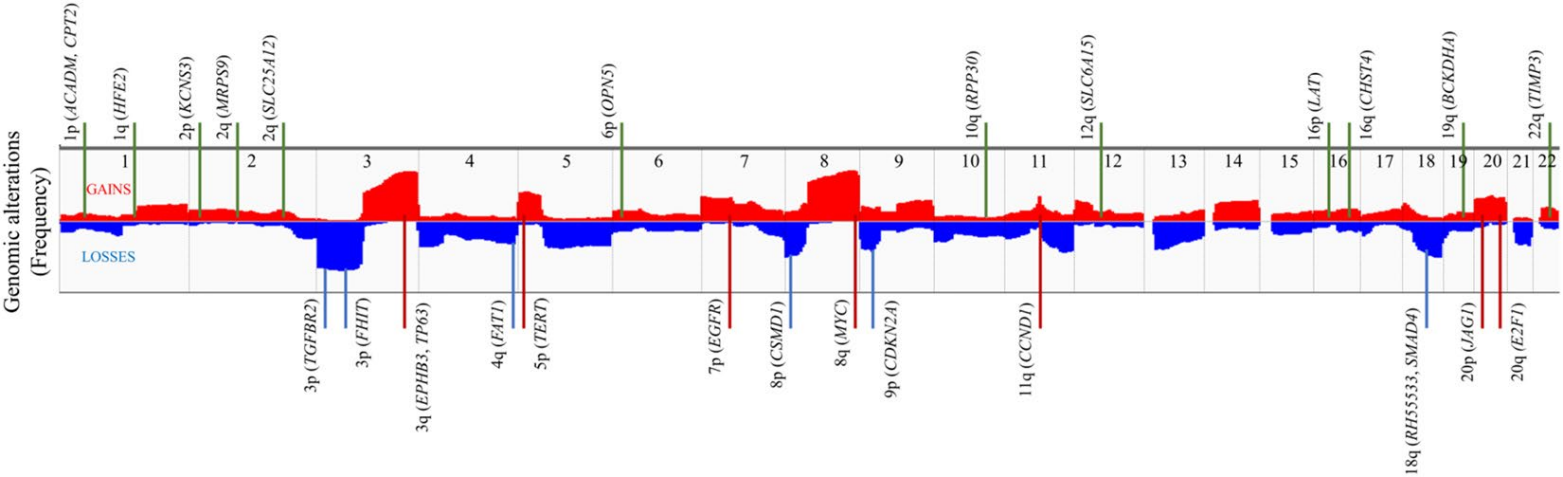
Selection of reference and target loci of the ddPCR assay. The middle panel illustrates the copy number gains (red, above the center line) and losses (blue, below the center line) of corresponding chromosomal regions (numerical numbered from left – chromosome 1 to right – chromosome 22, and separated by dashed line) identified from The Cancer Genome Atlas (TCGA) dataset. Selected reference (green line, above the middle panel of TCGA data) and target (blue for losses, and red for gains, below the middle panel of TCGA data) loci and their chromosome location are listed and mapped to TCGA data.

The performance of the ddPCR assay on clinical samples was further verified through application to DNA extracted from clinical FFPE tissue biopsy blocks previously studied by array CGH^32^. Five samples were selected representing different histopathological stages, including 2 LGDs (Oral 67 and 48), 1 HGD (Oral 44), and 2 OSCCs (Oral 75 and 91). In Oral 67, a non-progressing LGD, no recurrent gains were observed in any of the 24 target loci. However, the only statistically significant loss at the *TERTx2* locus at 5p observed, consistent with the log_2_ value obtained from array CGH data (Fig. 4A), was not generally associated with oral cancer progression risk (Fig. 5). In Oral 48, histopathologically classified as a progressing LGD, 17 of the 24 target loci showed statistically significant CNAs, including gains at 3q, 5p, 7p, 8q, 11q and 20p/q and losses at 4q, 8p, 9p and 18q (Fig. 4B). Collectively, these observed CNAs are consistent with CGH array data and with OSCC progression (Fig. 5). Significant CNAs were also detected in Oral 44 (HGD) and in the 2 OSCC samples (Oral 75 and 91) (Fig. 4C–E), with the ddPCR results again in good agreement with benchmark array CGH data for these samples (Fig. 4F and Table 1).

## Discussion

Although CNAs are an important event involved in tumorigenesis, their detection in clinical cancer genetics labs is generally limited to few cytogenetic-based assays focusing on gains of a single gene, such as *ERBB2* used to determine breast cancer patient’s suitability for targeted therapy^46^. In principle, technologies that screen the entire genome for CNAs, such as array CGH, SNP arrays, and WG-NGS, can ameliorate this problem, but are at present too costly and time intensive for general clinical use. Our novel multiplexed ddPCR assay is able to detect common recurring CNAs in DNA isolated from either oral cell lines or clinical samples of varying disease stage. The CNAs recorded are accurate, as shown through comparison to CGH, SNP arrays, and WG-NGS, all of which are considered gold-standards for genome-wide detection of copy number gains and losses^47^. These capabilities, when combined with the relatively low cost and fast turn-around time of the method, suggest that our multiplexed ddPCR assay possesses attributes needed for clinical translation.

The design of the ddPCR assay presented several challenges, including establishing a stable reference for CNA quantification, selection and precise positioning of target loci, and the ability to account and correct for fragmentation in DNA extracted from FFPE tissue^26^. By successfully addressing these challenges, the multiplexed ddPCR assay is able to identify several types of oncogenic events associated with OSCC progression, including HDs and high-level gains. By design, the ddPCR assay precisely targets regions with common recurrent gains or losses in oral cancer progression^7,10,48^. For example, the ddPCR assay detects recurrent HDs, such as at *CDKN2A*, that may go undetected by CGH array, and in some cases even SNP array, because of their submicroscopic size (<500 kbps). We have also demonstrated a means by which ddPCR data can be used to infer average ploidy level, which has not been done before. The ability to quantify high-level gains, which can be challenging to CGH or SNP arrays due to a combination of algorithm limitations and saturation of the signal^39,40^, could be clinically important. For instance, *CCND1* expression levels correlate to radio-sensitivity and therefore may be used to predict effectiveness of radiation therapy in OSCC^42^.

The novel multiplexed ddPCR assay described here demonstrate its ability to detect recurrent CNAs frequently gained or lost during oral carcinogenesis. While no significant CNAs in the 24 target loci were detected in either the normal oral cell line OKF4 E6E7 or the normal oral tissue and non-progressing LGD biopsies, a number of target loci were found to have significant gains or losses in the dysplastic and SCC oral cell lines, as well as in clinical oral biopsies classified as either progressing-LGD, HGD or SCC. In the dysplastic cell line POE9n tert, the observed pattern of CNAs is indicative of p16 (*CDKN2A*) inactivation and telomerase (*TERT*) activation; results of the assay are therefore consistent with an oncogenic pathway implicated in cellular immortalization and tumor progression^37^. In the progressing-LGD, loss of *CDKN2A* was observed along with other CNAs known to correlate with oral carcinogenesis^48^, including losses at *FAT1* and *CSMD1*, gains at *MYC* and 20p/q, and high-level gains at *EGFR*. Taken together, these results provide initial evidence that the pattern of common recurrent CNAs detected by ddPCR may be used to differentiate benign or noncancerous tissue from precancerous or cancerous tissue.

Although our primary goal was to establish a clinically-amenable method to detect CNAs as a predictor of cancer progression by targeting loci where gains or losses are indicative of oncogene activation or TSG suppression, respectively, there is growing evidence that CNAs in these regions also have prognostic value. For example, gains at *EGFR* or *CCND1*, and/or loss of 18q have been associated with lower survival and disease progression in OSCC^49^. Detection of high-risk HPV strains may also have prognostic value, as HNSCC patients with HPV-positive cancers have better outcomes and may have improved response to certain treatments^29,30^. Additionally, the identification of HPV positive tissue in lymph nodes may be used to localize the site of origin when the primary tumor site is not clinically apparent^28^.

In conclusion, the multiplexed ddPCR assay we have reported on here demonstrate its clinical value in detecting multiple target loci for common recurrent CNAs known to be associated with oral cancer progression. Further study of this assay using a larger cohort of progressing and non-progressing oral lesions is on-going to examine its ability to assess the oral cancer progression risk of histologically similar low-grade lesions.

## Methods

### Selection of reference loci to establish CNA-neutral benchmark

Thirteen reference loci (Fig. 5, Supplementary Table S1) were identified from chromosomal regions (1p, 1q, 2p, 2q, 6p, 10q, 12q, 16p, 16q, 19p, 22q) found to have low CNA frequency rates in TCGA data for HNSCC. Additionally, for one reference loci (*CPT2*), amplification templates of various lengths were designed to correct any bias to measured CNAs arising from significant fragmentation of the DNA isolated from a sample. The algorithm used to make those length-based corrections, as well as that used to define a CNA-neutral benchmark from ddPCR data for the 13 reference loci, has been described previously^26^.

### Selection of target loci for detection of common CNAs in OSCC

A total of 24 target loci (Fig. 5, Supplementary Table S1) were designed to query twelve chromosomal regions, including 3p, 3q, 4q, 5p, 7p, 8p, 8q, 9p, 11q, 18q, 20p, and 20q, where recurrent CNAs have been observed and correlated with oral cancer progression from oral premalignant lesions^48^, and/or where TCGA data have revealed frequent gains (3q, 5p, 7p, 8q, 11q, and 20p/q), losses (3p, 4q, 8p and 18q) or both (9p)^8^. Chromosomal regions associated with focal CNAs are queried with proximal target loci (*e.g. CDKN2Ai1* and *CDKN2Ax3* for the *CDKN2A* gene at 9p21.3), while broad regions are queried with distal target loci (*e.g. EPHB3x3* and *TP63i2* for the sub-telomeric region of 3q).

### Primers and probes used for ddPCR amplification of reference and target loci

HPLC purified probes dual labeled with 3′-IBHQ and either 5′-FAM or 5′-HEX, as well as all standard desalted primers, were purchased from IDT Inc. (Coralville, IA). Sequences for the primers and probes for reference and target loci are shown in Supplementary Table S1. The primers and probes used to detect and quantify the E6 region of HPV types 16 and 18 were previously described^27^. All primers and probes were resuspended to 100 µM in IDTE (10 mM Tris, pH 8.0, 0.1 mM EDTA) buffer.

### Cell lines

The cell lines OKF4 E6E7, POE9n tert, CAL27, SCC-4, SCC-9, SCC-25, SiHa, and HeLa were obtained from the B.C. Cancer Research Centre with their original sources and culture methods reported in previous CGH studies^31,33,34^. The identity of the cell lines was confirmed by CGH arrays^31,33,34^, SNP arrays^35^ and/or WG-NGS^36^.

### Clinical samples

A total of 11 clinical samples were analyzed. Four normal blood samples from healthy volunteers, one non-diseased frozen, and six FFPE tissue samples (one normal, two LGL, one HGL, and 2 OSCC)^32^ were collected under approval from the UBC/BCCA Clinical Ethics Research Board (H15-01554).

### DNA sample preparation

DNA extraction protocols used on buffy coat from blood samples, and on micro-dissected frozen and FFPE tissue samples were described previously^26^. For cell lines, cultured cells were trypsinized and harvested, then washed with 1× PBS, centrifuged, and resuspended in lysis buffer. DNA was extracted from the digested tissue using a SQ blood DNA kit (D5032-00, Omega Bio-tek Inc., Norcross, GA) according to the manufacturer’s instructions. All the DNA samples were quantified using a Qubit 2.0 Fluorometer (ThermoFisher Scientific, Waltham, MA).

### ddPCR experiments

All experiments were performed using a QX200 droplet generator and reader (Bio-Rad Inc., Hercules, CA). Supplementary Figure S1 shows the schematic representation for ddPCR. The multiplexed ddPCR assay uses a total of 16 unique 4-plex amplification reactions, performed in duplicate in a total of 32 reaction wells. The four reactions performed in each well are provided in Supplementary Table S2. Each 4-plex ddPCR 20 μl reaction was prepared with 10 μl of 2X ddPCR Supermix for probes (No dUTP) (Bio-Rad Inc.), forward and reverse primers, each at a final concentration (*C*_*t*_) = 900 nM, and a combination of FAM and HEX-labeled probes at a *C*_*t*_ = 60 to 300 nM (Table S2) so as to obtain a staggered layout of target-positive droplet clusters in the ddPCR output^26^. For each of the samples tested, a total of ~300 ng (~10 ng/well) of DNA was used. Further details of the protocol used in the ddPCR workflow have been provided previously^26^.

### Analysis of ddPCR output

Our algorithm for analyzing assay output to quantify CNAs has been previously described in detail^26^. Briefly, droplet counts are converted to a copy number ratio, *R*_*i/cr*_, for each reference or target loci *i*, together with the associated error (standard deviation) *σ*_*Ri*/*cr*_ in the value (where, *i* = the concentration of the reference or target locus, and *cr* = the concentration of the constant reference locus *HFE2* that is present in all 32 of the 4-plex ddPCR reaction wells). As we have previously described^26^, *R*_*i/cr*_ values, determined for three amplicons of unique lengths (89, 106 and 125 bp) at the *i* = *CPT2* locus, are used to analyze if fragmentation of the DNA isolated from the sample is significant. For those samples determined to have statistically significant fragmentation, the *R*_*i/cr*_ values for reference and target loci are corrected based on their amplicon lengths as previously described^26^. For the ddPCR assay reported here, the amplicon lengths for the set of reference (97 to 106 bp) and target loci (93 to 124 bp) have been specifically designed to be short and similar in size to minimize the fragmentation corrections required (as well as the uncertainty (*σ*_*Ri*/*cr*_) associated with *R*_*i/cr*_) as the relative amount of amplifiable DNA for larger amplicons can be appreciably lower in samples where fragmentation is significant^26^. The *R*_*i/cr*_ values for reference loci are then analyzed by a k-means type cluster analysis to select those reference loci inferred to be CNA-neutral. Their *R*_*i/cr*_ values are averaged to establish a benchmark (*b*). Normalized *R*_*i/b*_ $({R}_{i/b}^{Norm})$ are then determined for each target locus. Statistically significant CNAs for each target locus are identified using the null hypothesis for a chosen confidence interval (CI). Here we selected a CI = 99%, ensuring that significant CNAs are identified with high confidence. Gains and losses are identified as ${R}_{i/b}^{Norm} > 0\,{\rm{and}}\, < 0$, respectively, with high-level gains and homozygous deletions (HD) identified as ${R}_{(i/b)}^{Norm} > 1\,{\rm{and}}=-1$, respectively.

### Algorithm to infer ploidy level

In homozygous systems, ${R}_{i/b}^{Norm}$ values are expected to represent discrete changes in copy numbers at each target locus *i*. Thus, in a pure diploid cell line low-level CNAs ($-1.0 < {R}_{i/b}^{Norm} < 1.0$) should be identified by an ${R}_{i/b}^{Norm}=\pm 0.5$, while in a triploid cell line, low-level CNAs should carry a ${R}_{i/b}^{Norm}=\pm 0.33\,{\rm{or}}\,\pm 0.66$. Based on this, ${R}_{i/b}^{Norm}$ values determined in our ddPCR assay may be used to infer ploidy level. Briefly, for a given sample, measured target-loci ${R}_{i/b}^{Norm}$ values between −0.99 and 0.99 (representative of low-level gains in a sample) are compared to the expected discrete copy number changes for an assumed ploidy (*R*_*p*(*L)*_), where *L* represents the ploidy level being queried (ranging from 1 to 5 genomic copies in the algorithm used here). In this analysis, we exclude high-level gains (due to larger values of σ_*R*_), as well as HDs, as both are non-informative with respect to ploidy level (*L*). For each assumed *L* being queried by the algorithm, the total number of target loci (*N*_*t(L)*_) for which

1 $${R}_{p(L)}={R}_{i/b}^{Norm}\pm z{\sigma }_{R}$$

is determined at the *z* for the chosen CI (99% in our analysis). Note that as the assumed ploidy level is increased, the value of *R*_*p(L)*_ will decrease (0.5 for diploid, 0.33 for triploid, 0.25 tetraploid), increasing the odds that the number of target loci with ${R}_{i/b}^{Norm}$ that match the chosen *R*_*p(L)*_ will increase. To account for this bias, we determine the average and associated error (*N*_t(L)_* ± *σ*_N_*) of the total number of target loci expected to match each ploidy level due to random chance using a Monte Carlo simulation; the details of that simulation are provided in the Supplementary Methods. The alternative hypothesis H: *N*_*t(L)*_ > *N*_t(L)_* is then tested for each assumed ploidy level using the relation

2 $${z}_{L}=\frac{{N}_{t(L)}-{N}_{t(L)}\ast }{{\sigma }_{N}\ast }$$

The inferred ploidy level is given by the *L* that maximizes *z*_*L*_, with the level of significance determined from the associated analysis.

### Array CGH data

The set of three array CGH probes that overlay or lie adjacent to each target locus *i* were mapped using the UCSC Genome Browser NCBI35/hg17 assembly^50^. The log_2_ values for the array CGH data^31–34^ for each of those probes were calculated and averaged. The resulting log_2_ values were then rescaled to facilitate direct comparison with ddPCR-derived ${R}_{i/b}^{Norm}$ values as follows; HD and high-level gains, typically defined as regions with log_2_ values of −0.8 and 0.8 respectively^33^, were taken as log_2_ value minima/maxima and used to rescale CGH data to corresponding range of ddPCR-determined ${R}_{i/b}^{Norm}$ values (−1.0 for HD and +1.0 for high-level gain).

### SNP array data

SNP-array based copy number analyses of several of the cell lines investigated have been performed previously, with results archived by the COSMIC (Catalogue of Somatic Mutations in Cancer) cell line project^51^ and data from the Affymetrix SNP 6.0 array analyzed with PICNIC (Predict Integral Copy Numbers In Cancer) software^52^. Inferred copy number of chromosomal segments and the genome average copy number for each cell line are available through canSAR (cansar.icr.ac.uk)^35^. To allow for comparison between ddPCR and CGH array data, the change (Δ) in copy number from SNP data was determined for the different target loci by subtracting the inferred ploidy level (integer rounded average genomic copy number) from the copy number assigned to the chromosomal segment representing the target locus.

### WG-NGS data

WG-NGS data for HeLa cell line have been previously published^36^. The ploidy levels of chromosomal regions of interest were approximated based on the freely accessible figures and supplemental files published.

## Electronic supplementary material

Supplemental information
