# Supplementary material for: Detection of clinically relevant copy number alterations in oral cancer progression using multiplexed droplet digital PCR
Source: Sci Rep. 2017 Sep 19;7:11855. doi: 10.1038/s41598-017-11201-4 (PMC5605662; doi:10.1038/s41598-017-11201-4)
Supplement: Supplementary file 1 — Supplemental information [file 41598_2017_11201_MOESM1_ESM.docx]

**Supplementary information for:**

**Detection of clinically relevant copy number alterations in oral cancer progression using multiplexed droplet digital PCR**

Curtis B. Hughesman^1,2,3^, X.J. David Lu^1,3^, Kelly Y.P. Liu^1,3^, Yuqi Zhu^1,3^, Rebecca M. Towle^3^, Charles Haynes^2,*^ & Catherine F. Poh^1,3,4,*^

*** Corresponding authors:**

Catherine F. Poh

email: [cpoh@dentistry.ubc.ca](mailto:cpoh@dentistry.ubc.ca)

Charles Haynes

email: [israels@chbe.ubc.ca](mailto:israels@chbe.ubc.ca)

**Table of contents:**

[Table S1. Primers and probes used in ddPCR experiments 2](#_Toc490812487)

[Table S2. Loci in the 16 unique 4-plex ddPCR 4](#_Toc490812488)

[Figure S1. Schematic representation of droplet digital PCR (ddPCR). 5](#_Toc490812489)

[Figure S2. Comparison of CNAs for 24 target loci in immortalized normal and dysplastic oral cell lines determined by ddPCR or CGH arrays. 6](#_Toc490812490)

[Figure S3. Quantification of viral load in HPV positive cell lines. 7](#_Toc490812491)

[Figure S4. CNA analysis for 24 target loci in the SiHa cell lines determined by ddPCR CGH, or SNP array. 8](#_Toc490812492)

[Figure S5. CNA analysis for 24 target loci determined in the HeLa cell line by ddPCR, CGH array, SNP array or WG-NGS. 9](#_Toc490812493)

[Figure S6. CNA analysis for 24 target loci determined by ddPCR in normal blood or tissue. 10](#_Toc490812494)

[Supplementary Methods: Monte Carlo simulation to determine N_t(L)_* and σ_Nt(L)_* 11](#_Toc490812495)

# Table S1. Primers and probes used in ddPCR experiments

| **Chromosomal region** | **Locus** | ***l^1^* (bp)** | **Forward Primer*^2^*** | **Reverse Primer^2^** | **Probe*^2,3^*** |
| --- | --- | --- | --- | --- | --- |
| **Reference Loci** | | |  |  |  |
| 1q21.1 | *HFE2* | 97 | gggatccagtttgtcgattca | agctgtctgccgaatgattatag | aactgctaaccctgggaaccatgt |
| 1p32.2 | *CPT2(89)* | 89 | gcccagcagtgaacctt | gcagcctatccagttgtcat | cctgtggtctctgatggctttggt |
| 1p32.3 | *CPT2(106)* | 106 | ccagcagtgaaccttggg | ctgggtaggaagagacattgc | cctgtggtctctgatggctttggt |
| 1p32.3 | *CPT2(125)* | 125 | tacgggcagataaaccacaa | cagttgtcatgaacagcatacc | cctgtggtctctgatggctttggt |
| 2p24.2 | *KCNS3* | 101 | gcttatcgctatctcctccttg | catccacttctccatcctcatt | cgttcacagcatgtcggagttcca |
| 1p31.1 | *ACADM* | 101 | gatgttcagatactagaggaattgt | agctcccattgcaacttt | ttaattggtgacggagctggtttc |
| 2q12.1 | *MRPS9* | 100 | aacgtgaaacttacacagagga | gtttctggatcttctcccatca | ctctttcctatgttgaactcttcaatctgc |
| 2q31.1 | *SLC25A12* | 106 | gatcacatcggtggatacagac | gaaccacagcaacactagga | tttcgatgcctgcaaacgtggc |
| 6p24.2 | *OPN5* | 101 | agaaagcacgcctacatctg | aagggctcaggtacgtagt | ctgggcctatgcttccttctggac |
| 10q23.31 | *RPP30* | 98 | gggaaggaagtatgacagatgtt | gaagccatccttgagtccttag | agcaaagtacaacaggaagacaccttgg |
| 12q12.31 | *SLC6A15* | 98 | tgttcgtcgcttcaaccttat | cctctaagttcacaggctctttc | acccttcctctcttataggtcacagatgc |
| 16p11.2 | *LAT* | 97 | cgtcctggactaggctga | aggtagcctgggttgtgata | cccagaaccagcctgtgaggat |
| 16q22.2 | *CHST4* | 99 | cttcagacctgggtgcataac | ccaagcctgggagacattaag | tgaccacgctttccacacaaatgc |
| 19q13.2 | *BCKDHA* | 105 | caccagtgacgacagttcag | ccttggctcagcagatagtg | ccgctcggtggatgaggtcaatta |
| 22q12.3 | *TIMP3* | 105 | gcgtctatgatggcaagatgta | ccaggtgataccgatagttcag | ctgtgcaacttcgtggagaggtgg |
| **Target Loci for CNA detection** | | | |  |  |
| 3p24.1 | *TGFBR2i6* | 103 | gagcccttcttggagttgtg | gacttccacatccctagcattta | tacttgttcagctggctgctacgt |
| 3p14.2 | *FHITi5* | 105 | agactattctgtgacatttgagca | gtttattcttcgtggaatgccaat | tgctatgttcactcaactccttcagacc |
| 3p14.2 | *FHITx5* | 108 | gtgaggacatgtcgttcagatt | ctggtaccacaggtttcctattc | agagggcttgatgagatgttggcc |
| 3p14.2 | *FHITi4* | 99 | gggtttttaattatacctctttgtagc | aagataaggatgcatattgttagtcc | aca+Caca+Cacaca+Cac |
| 3q27.1 | *EPHB3x3* | 97 | gtggagctcaagttcactgt | tgtcagcctcgtagtagaaga | cgtgactgcaacagcatcccc |
| 3q28 | *TP63i2* | 94 | tccgttatttcctgcatcctac | caagcagttcaaatccaaccc | tctgaggagggcagaggtcatgat |
| 4q35.2 | *FAT1x28* | 102 | ctgccggacatacaagagtt | tccagggtaatagtccgtatcg | tgtactcagcagatccaaacgcca |
| 4q35.2 | *FAT1i2* | 123 | taaacaaattgagccccacc | gactgcaggagtaaatgagcg | tccactatcattctcagagggcagga |
| 5p15.33 | *TERTi14* | 93 | cctgagcttaacagcttctactt | gcaggagtcacgacagaaat | tggaaatttcacctggagaagccga |
| 5p15.33 | *TERTx2* | 124 | gaccaagcacttcctctactc | ggaacccagaaagatggtctc | agagagctgagtaggaaggagggc |
| 7p11.2 | *EGFRi1* | 110 | ggactcttgagcggaagc | agaaagaaagtctccgggttt | tgaggagaagtttgctgtgagccc |
| 7p11.2 | *EGFRx28* | 100 | cattagctcttagacccacagac | caaaggaatgcaacttcccaaa | tgcaacgtttacaccgactagcca |
| 8p23.2 | *CSMD1i5* | 100 | ggtgttgcttcttccaggtaata | gctgaggacctgatttctacac | acacaggtgacccatttcgtagga |
| 8p23.2 | *CSMD1x5* | 93 | cccttcagagtacgagaacaac | agctgaaagtcagtgaagacc | cggactgcacctggaccattct |
| 8q24.21 | *MYCi1* | 98 | taagactaccctttcgagatttctg | cccagagagcaattaacacaataaa | atatattcacgctgactcccggcc |
| 8q24.21 | *MYCx2* | 94 | gagacatggtgaaccagagtt | gagaagccgctccacatac | ccggacgacgagaccttcatcaaa |
| 9p21.3 | *CDKN2Ax3* | 104 | tgtgggcatttcttgcga | ctcaagagaagccagtaaccc | ccggaagctgtcgacttcatgaca |
| 9p21.3 | *CDKN2Ai1* | 98 | ccaacgcaccgaatagttacg | aaacttcgtcctccagagtcg | atccaggtgggtagagggtctgca |
| 11q13.3 | *CCND1i3* | 97 | catgcgatgtcccttcagaata | gatgtcgcttctatgaccctaac | agcagcagggattgcagacaagtc |
| 11q13.3 | *CCND1x5* | 113 | gaggatgttcataaggccagta | ctgtaacatcaaaggcagaagg | aca+Caca+Cacaca+Cac |
| 18q21.1 | *RH55533* | 105 | cctccattgaaaagcttagtagc | gaggggcctagacagtgaca | agtccctttgacaccaccctgttg |
| 18q21.2 | *SMAD4x5* | 95 | tcaagtatgatggtgaaggatgaa | ggatgctggatggtttgaattg | tgactttgagggacagccatcgtt |
| 20p12.2 | *JAG1x23* | 101 | gaccagtgcttcgtccac | tcctggtaataggagtcagagg | cgagtgtcggtcttccagtctcca |
| 20q11.22 | *E2F1x7* | 101 | aggagttcatcagcctttcc | cccaaagtcacagtcgaaga | tcgactaccacttcggcctcga |
| **Additional target loci used for HD mapping** | | | | | |
| 3p14.2 | *FHITx6* | 93 | atgacctgcgtcctgatgaa | tgagagaggtcccatggaaatg | tctgggtcgtctgaaacaaatcggc |
|  | *D3S1234* | 111 | cctgtgagacaaagcaagac | gacattaggcacagggctaa | aca+Caca+Cacaca+Cac |
|  | *D3S1300* | 247 | agctcacattctagtcagcct | gaatgccaattccccagatg | aca+Caca+Cacaca+Cac |
| 9p21.3 | *D9S1749M* | 120 | agagggtacgcttgcaaat | ggtgcgggtgcagataa | aca+Caca+Cacaca+Cac |
|  | *CDKN2Ax1* | 120 | ggagccttcggctgact | atcggcctccgaccgtaa | tattcggtgcgttgggcagc |
|  | *D9S1748* | 125 | cacctcagaagtcagtgagt | gtgcttgaaatacacctttcc | aca+Caca+Cacaca+Cac |
|  | *D9S1814M* | 103 | accatggttcttctactcagga | ctgaccttctgtggcaattct | aca+Caca+Cacaca+Cac |

*^1^* Amplicon lengths determined from primer blast ([www.ncbi.nlm.nih.gov/tools/primer-blast/](http://www.ncbi.nlm.nih.gov/tools/primer-blast/)) and/or from the UCSC genome browser (<https://genome.ucsc.edu/>). For microsatellite biomarkers with a reported size range, the amplicon length used is the median of the range.

*^2^* Sequences reported in 5’ to 3’ direction. LNA bases are identified by capitals and a preceding + symbol.

*^3^* All DNA AND LNA probes were labelled with either 5’ FAM or 5’-HEX. DNA probes contained an internal ZEN quencher and a 3’-IBFQ, while LNA probes contained only a 3’-quencher (IBFQ or BHQ1).

# Table S2. Loci in the 16 unique 4-plex ddPCR

| **Reaction** | **Locus 1*** | **Locus 2*** | **Locus 3*** | **Locus 4*** |
| --- | --- | --- | --- | --- |
| *R1* | *ACADM* | *KCNS3* | *SLC25A12* | *HFE2* |
| *R2* | *BCKDHA* | *OPN5* | *CPT2(106)* | *HFE2* |
| *R3* | *CHST4* | *MRPS9* | *TIMP3* | *HFE2* |
| *R4* | *LAT* | *SLC6A15* | *RPP30* | *HFE2* |
| *R5* | *EGFRx28* | *CDKN2Ai1* | *HPV16* | *HFE2* |
| *R6* | *FHITi5* | *EPHB3x3* | *HPV18* | *HFE2* |
| *R7* | *TERTi14* | *FAT1x28* | *CPT2(89)* | *HFE2* |
| *R8* | *CSMD1i5* | *MYCi1* | *CPT2(106)* | *HFE2* |
| *R9* | *CCNDi3* | *SMAD4x5* | *CPT2(89)* | *HFE2* |
| *R10* | *JAG1x23* | *E2F1x7* | *CPT2(106)* | *HFE2* |
| *R11* | *FHITx5* | *D3S1481M* | *CPT2(106)* | *HFE2* |
| *R12* | *TP63i2* | *SJ107* | *CPT2(89)* | *HFE2* |
| *R13* | *RH47956* | *TERTx2* | *CPT2(125)* | *HFE2* |
| *R14* | *EGFRi1* | *CDKN2Ax3* | *CPT2(125)* | *HFE2* |
| *R15* | *CSMD1x5* | *MYCx2* | *CPT2(89)* | *HFE2* |
| *R16* | *RH55533* | *CCND1x5* | *CPT2(125)* | *HFE2* |

*A combination of 5’FAM and/or 5’HEX probes where used so as to segregate the droplet clusters in ddPCR output with the following concentration: Locus 1 = 300 nM FAM; Locus 2 = 140 nM FAM + 60 nM HEX; Locus 3 = 60 nM FAM + 140 nM HEX; Locus 4 = 300 nM HEX.


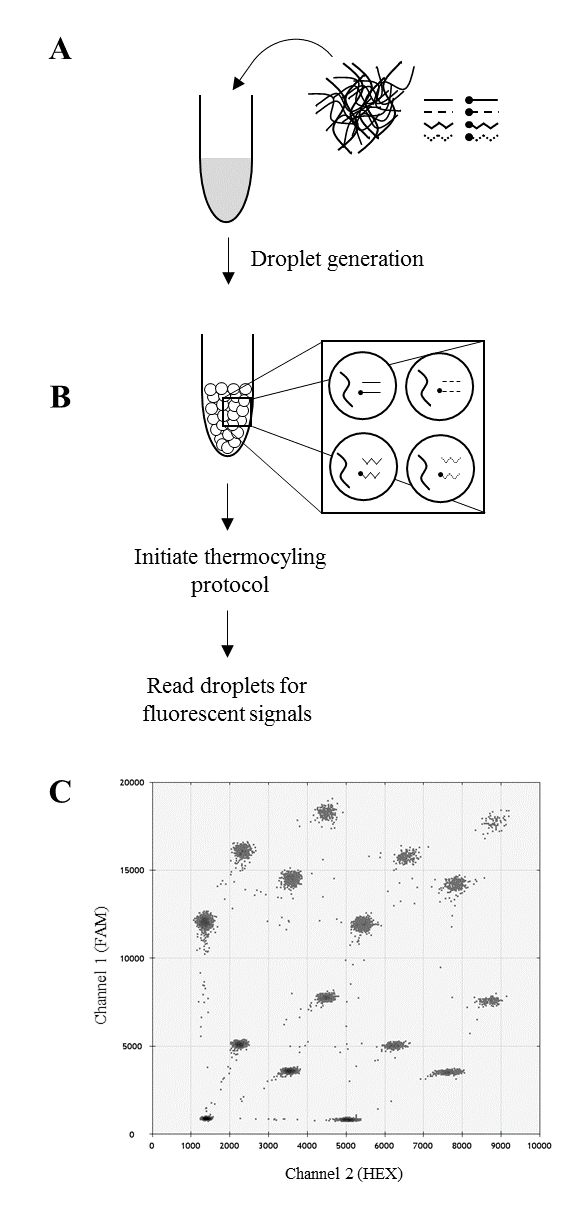


# Figure S1. Schematic representation of droplet digital PCR (ddPCR).

(**A**) Reactions contained ddPCR Supermix for probes (no dUTP) (Bio-Rad Inc., Hercules, CA), four sets of primers (dashed and solid, straight and zigzag lines each represent one set of primers) and their respective probes (lines with circles), and DNA templates (a mesh of squiggly lines). (**B**) Droplets were generated with oil and microfluidic system (Bio-Rad Inc., Hercules, CA). Theoretical partitioning of materials inside each droplet exemplified by four individual droplets. Following a thermocycling protocol, droplets were read for their fluorescent signals as exemplified in (**C**). Using a combination of FAM and HEX probes, the raw output of a ddPCR reaction with a staggered layout is observed.


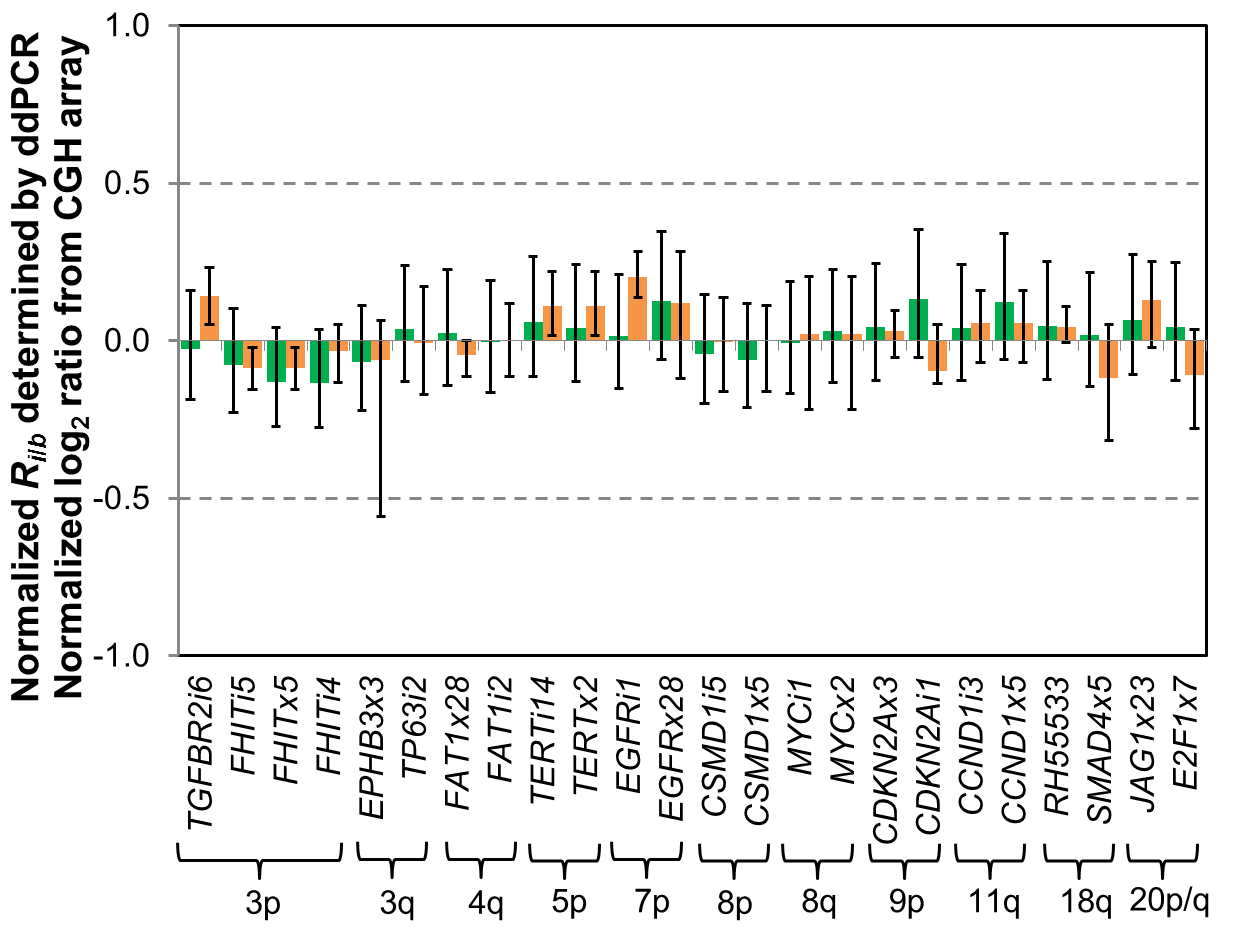


**A**


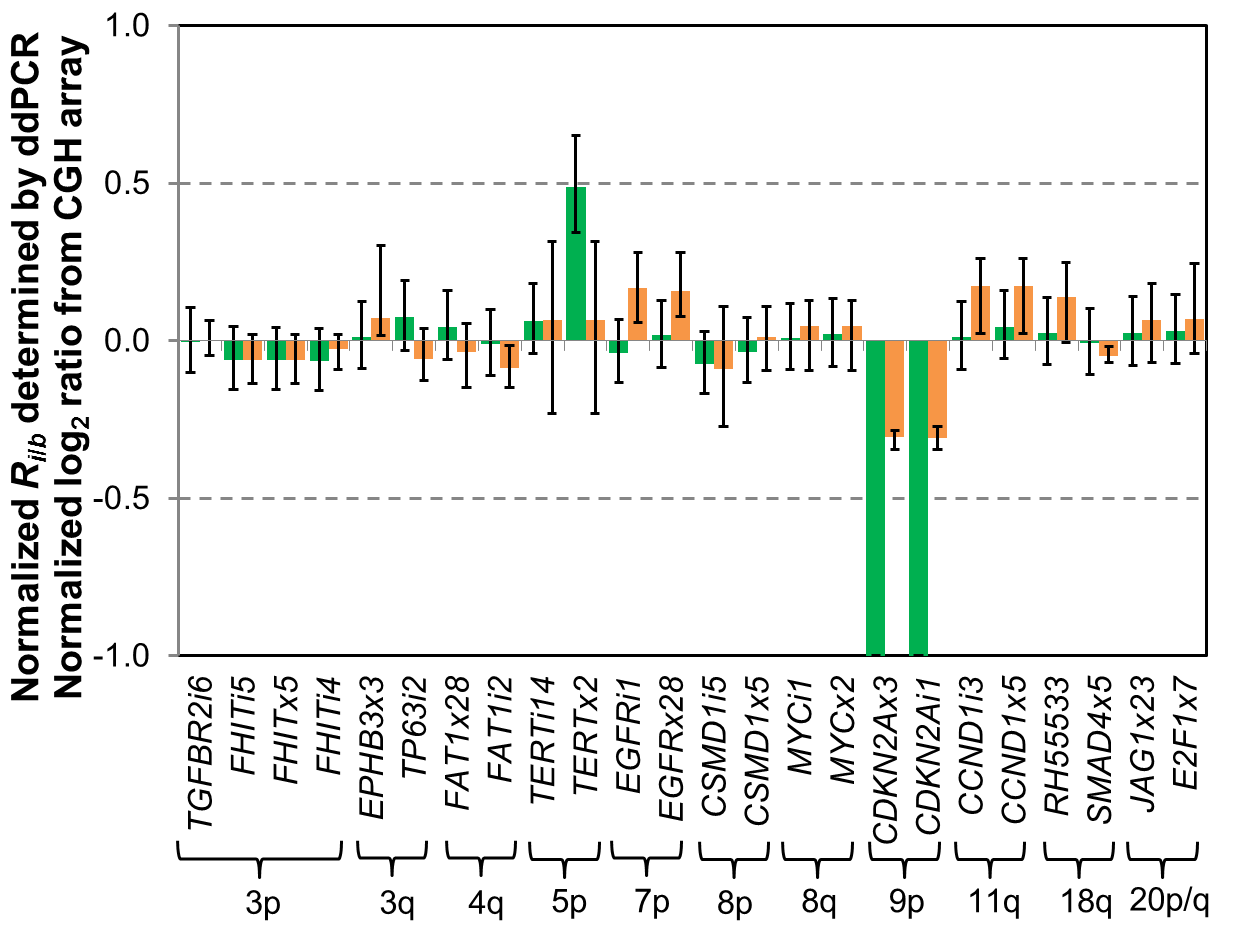


**B**

# Figure S2. Comparison of CNAs for 24 target loci in immortalized normal and dysplastic oral cell lines determined by ddPCR or CGH arrays.

CNAs determined by ddPCR (green bars) or CGH array (orange bars) for (**A**) normal oral cell line OKF4 E6E7 and (**B**) dysplastic oral cell line POE9n tert. Error bars for ddPCR data represent a 99% confidence interval while error bars for CGH show the respective high and low normalized log_2_ ratio for the probes that map closest to that target locus.

**
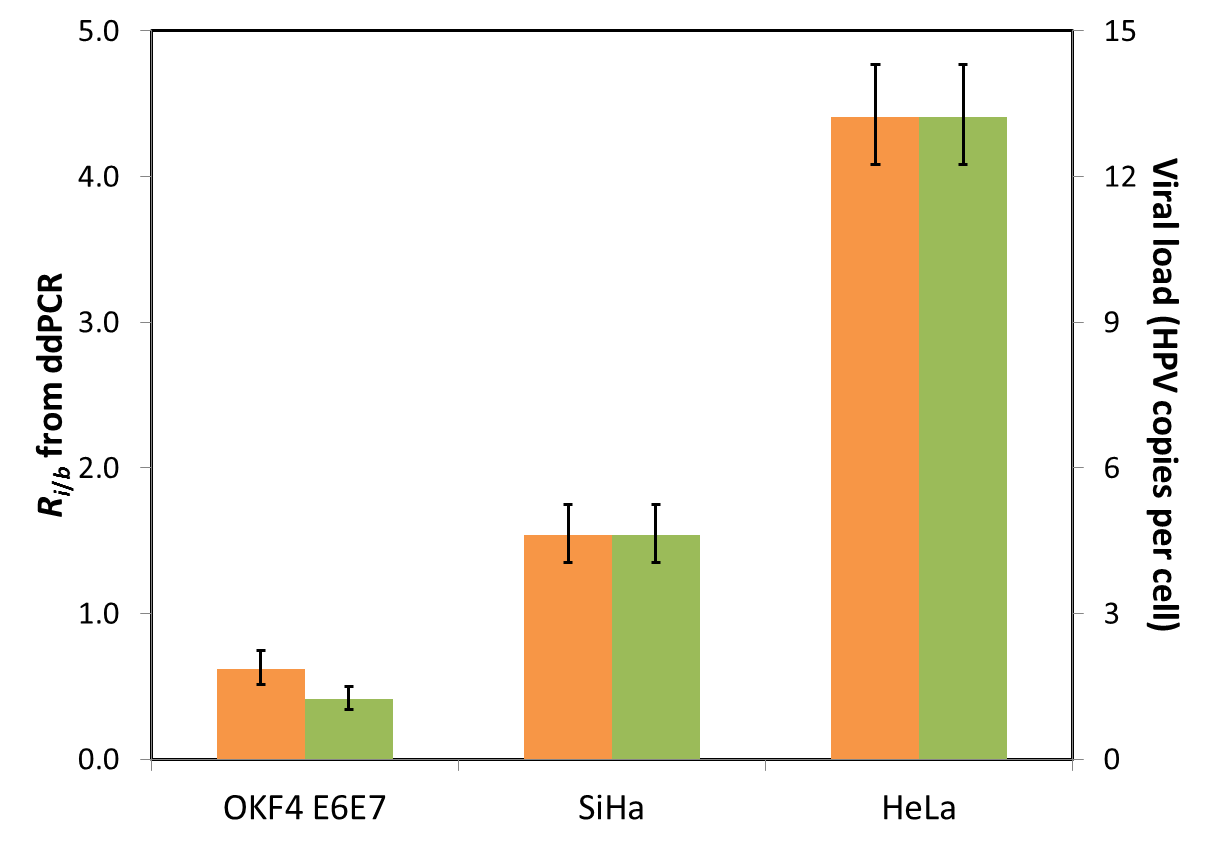
**

# Figure S3. Quantification of viral load in HPV positive cell lines.

The copy number ratio *R_i/b_* (orange bars) and the viral load (green bars) determined by ddPCR for the three HPV positive cell lines OKF4 E6E7 (HPV16+), SiHa (HPV16+), and HeLa (HPV18+). Viral load per cell was determined by multiplying *R_i/b_* (*i* = HPV16 or HPV 18) with the inferred ploidy of the cell line, which for OKF4 E6E7 was assumed to be diploid (*L* = 2), and for SiHa and HeLa was inferred by the alogirthm to be triploid (*L* = 3). Error bars represent a 99% confidence interval.


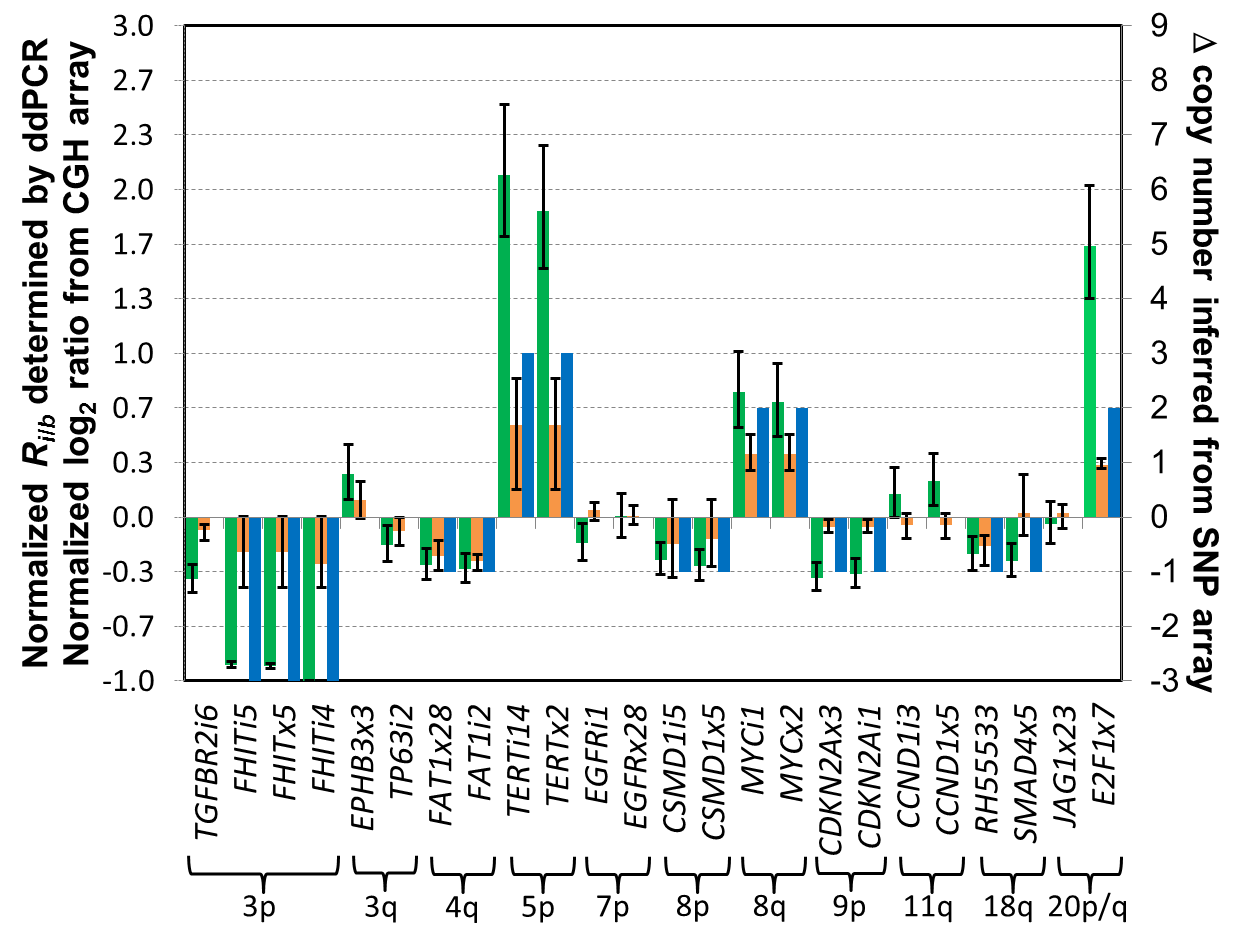


# Figure S4. CNA analysis for 24 target loci in the SiHa cell lines determined by ddPCR CGH, or SNP array.

CNAs determined by ddPCR (green bars), CGH (orange bars) or SNP array (blue bars). Error bars for ddPCR data represent a 99% confidence interval while error bars for CGH show the respective high and low normalized log_2_ ratio for the probes that map closest to that target locus. On the secondary y-axis the scale for the change in copy number inferred from SNP array for each cell line was set to match the inferred ploidy level determined by ddPCR which for the SiHa was triploid (*L*=3).


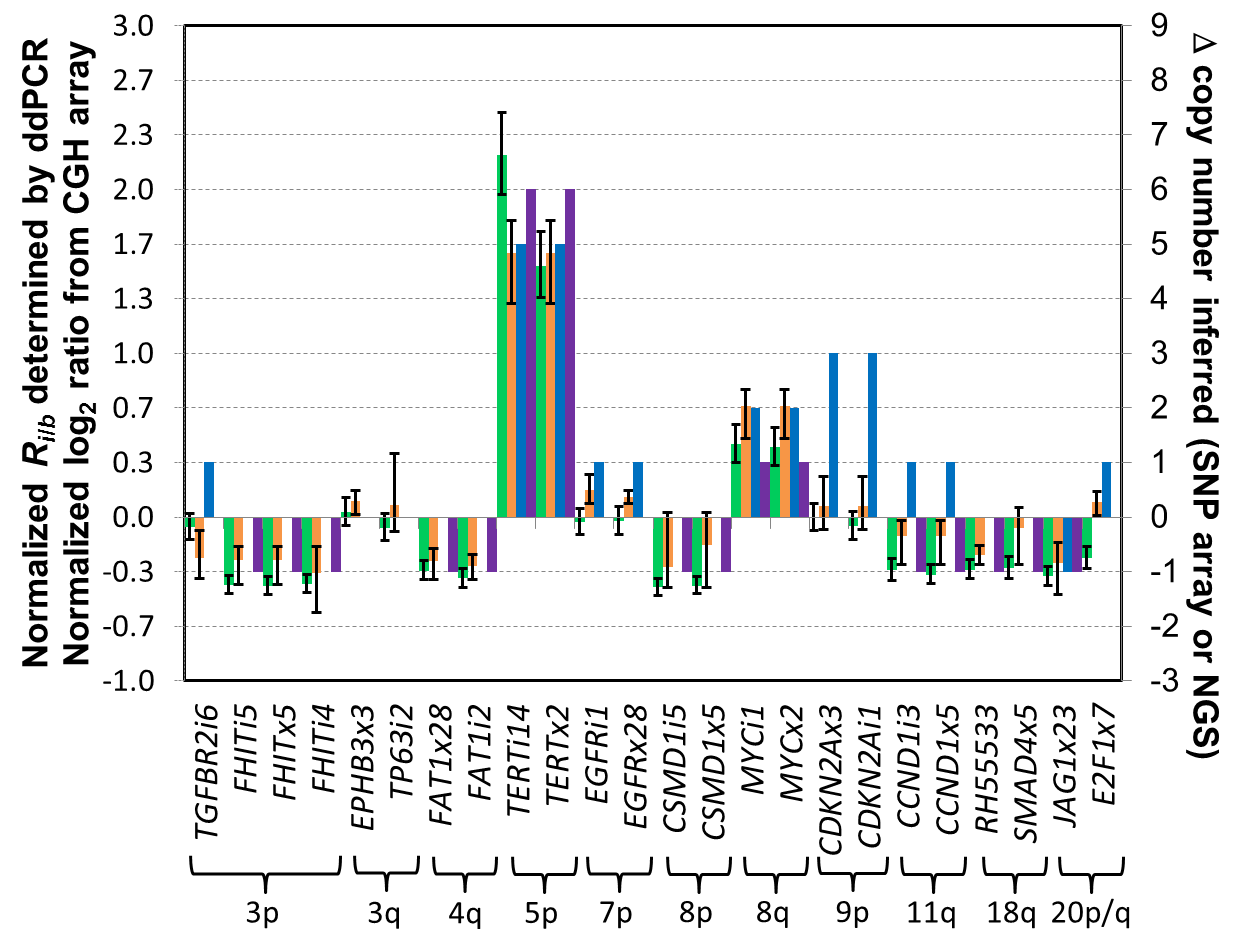


# Figure S5. CNA analysis for 24 target loci determined in the HeLa cell line by ddPCR, CGH array, SNP array or WG-NGS.

CNAs determined by ddPCR (green bars), CGH (orange bars), SNP array (blue bars) or WG-NGS (purple bars). Error bars for ddPCR data represent a 99% confidence interval while error bars for CGH show the respective high and low normalized log_2_ ratio for the probes that map closest to that target locus. On the secondary y-axis the scale for the change in copy number inferred from SNP array or NGS for each cell line was set to match the inferred ploidy level determined by ddPCR which for the HeLa was triploid (*L*=3).


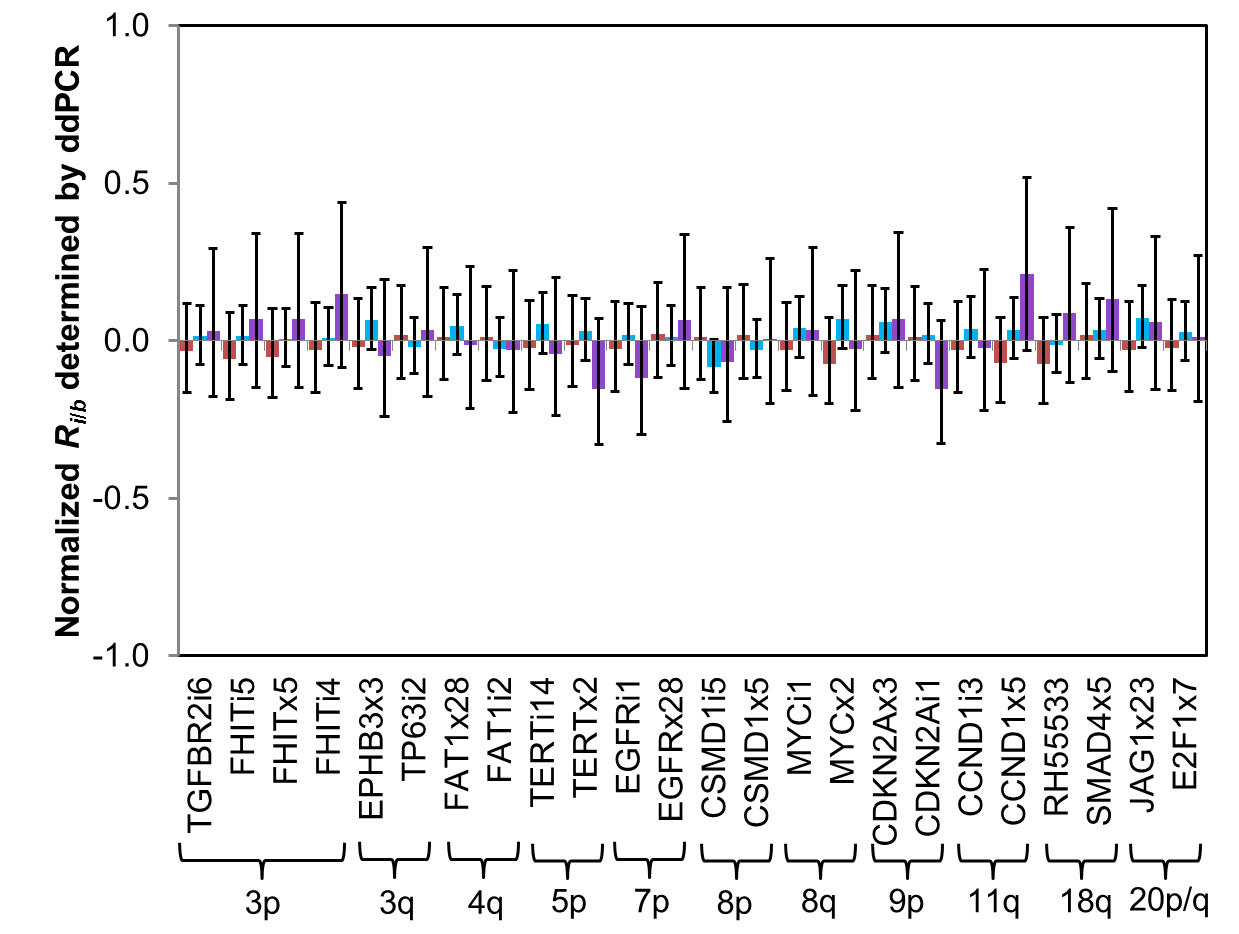


# Figure S6. CNA analysis for 24 target loci determined by ddPCR in normal blood or tissue.

CNAs determined by the multiplexed ddPCR assay in DNA extracted from normal blood (red bars), normal non-diseased frozen tissue (blue bars) and normal non-diseased FFPE tissue block (purple bars). Error bars for ddPCR data represent a 99% confidence interval.

# Supplementary Methods: Monte Carlo simulation to determine *N_t(L)_** and σ*_Nt(L)_**

As ploidy level (*L*) increases, the size of the partition that represents discrete changes in copy number (*R_p_*_(_*_L)_*) decreases. Thus the expected number of target loci (***N_t(L)_**)** with low level CNAs (normalized *R_i/b_* > -1.0 or < 1.0) determined to be statistically equivalent to a *R_p_*_(_*_L)_* will increase simply due to chance. To account for this we used a Monte Carlo simulation to determine the average *N_t(L)_** and the associated error (standard deviation) σ*_Nt(L)_**. In this simulation, each target locus was randomly assigned a *R_i/b_* value between = -0.99 and 0.99 (using a uniform distribution) and an assumed error (99% CI) was calculated as *z*σ*_R_* = 0.1 × *R_i/b_* (non-normalized). Using Equation 1 we could then estimate *N_t(L)_** for each value of *L* queried (1, 2, 3, 4 or 5). This step was repeated 1000 times allowing us to determine the average *N_t(L)_** and the error σ*_Nt(L)_**. Using this approach, a separate Monte Carlo simulation was performed for each of the cell lines, with the total number of target loci that were assigned random *R_i/b_* values being set to match the number of informative (low-level CNAs) target loci for that cell line.
